# A Young Patient Presenting with Dilated Cardiomyopathy and Renal Infarction during Treatment with Isotretinoin: Mere Coincidence or Serious Side Effect of a Drug Commonly Used in Adolescence?

**DOI:** 10.3390/diagnostics13091543

**Published:** 2023-04-25

**Authors:** Martino Pepe, Gianluigi Napoli, Maria Cristina Carella, Daniele De Feo, Rocco Tritto, Andrea Igoren Guaricci, Cinzia Forleo, Marco Matteo Ciccone

**Affiliations:** 1 Cardiovascular Diseases Section, Interdisciplinary Department of Medicine (DIM), University of Bari Aldo Moro, Piazza G. Cesare 11, 70124 Bari, Italymarcomatteo.ciccone@gmail.com (M.M.C.); 2 Department of Precision and Regenerative Medicine and Ionian Area (DiMePRe-J), University of Bari Aldo Moro, Piazza G. Cesare 11, 70124 Bari, Italy

**Keywords:** acute heart failure, cardiac transplant, thrombosis, cardiomyopathy, side effect

## Abstract

**Highlights:**

**What are the main findings?**

- We presented a case of new onset dilated cardiomyopathy (DCM) with renal infarction in a young man on a treatment with high-dose isotretinoin.
- Possible known causes of DCM using a complete cardiac imaging assessment, genetic testing, and laboratory analysis have been ruled out.

**What is the implication of the main finding?**

- The possible association of DCM with isotretinoin assumption is, to the authors’ opinion, deserving of further investigations.

**Abstract:**

Isotretinoin or 13-cis-retinoic acid (RA) is one of the most effective and widely used drugs for the treatment of severe acne vulgaris. Despite being deemed safe, no definite consensus has been reached on the cardiovascular risk of RA derivatives. We report a case of heart failure due to dilated cardiomyopathy (DCM) and concomitant renal infarction occurring after 5 months of isotretinoin use in a previously healthy 18-year-old male. The patient, with a history of acne vulgaris, presented to our emergency department with left iliac fossa pain and effort dyspnea. A trans-thoracic echocardiogram showed DCM and severely reduced left ventricle ejection fraction (LVEF: 29%). During hospitalization, a total body computed tomography (CT) showed an ischemic lesion in the left kidney. Ischemic, autoimmune, infective, and heritable causes of DCM were ruled out. Cardiac magnetic resonance (CMR) evidenced LV circumferential mid-wall late gadolinium enhancement. Heart failure therapy was promptly started and up-titrated, but only poor LVEF improvement was detected overtime. Our case aims to raise awareness on rare life-threatening cardiovascular events possibly associated with isotretinoin use. To the best of our knowledge, this is the first described case of renal thromboembolism and severe DCM leading to implantable cardioverter-defibrillator (ICD) implantation occurring during isotretinoin treatment.

**Figure 1:**
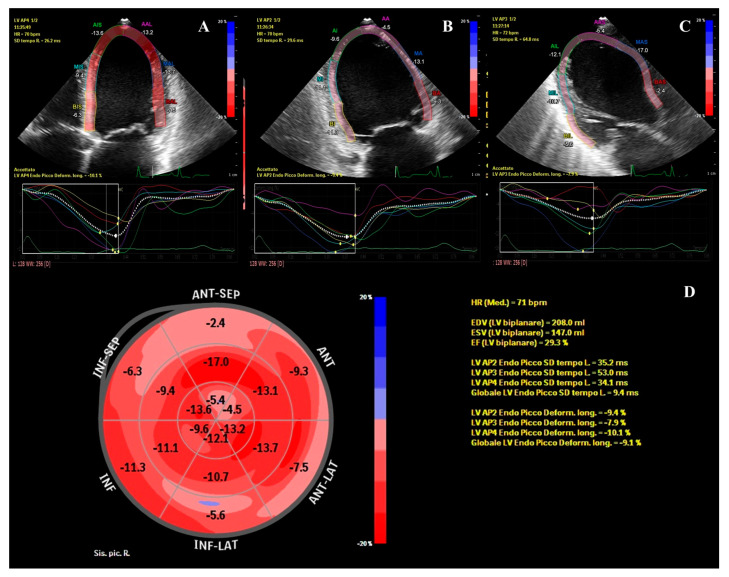
Left ventricular global longitudinal strain (GLS) assessment with two-dimensional speckle-tracking echocardiography. The figure shows the analysis of left ventricular GLS from the 4-chamber (**A**), 2-chamber (**B**), and 3-chamber (**C**) views, and the respective time-to-strain curves (**D**). The polar map with the regional values and the global longitudinal strain is also depicted; GLS = − 9.1%; LVEF = 29.3%. These echocardiographic data refer to a previously healthy 18-year-old male who accessed our emergency department (ED) in August 2021 for left iliac fossa pain and worsening dyspnea on exertion. In the ED, a computed tomography (CT) scan revealed a left renal infarction and bilateral pleural and peritoneal effusions. After echocardiographic evidence of left ventricular (LV) dilatation and severely reduced LV ejection fraction (EF), the patient was transferred to our cardiology unit. He denied cardiovascular risk factors and family history of cardiovascular disease or sudden death. Anamnesis was negative for febrile episodes and/or other symptoms of infectious disease over the previous weeks. Isotretinoin (also known as 13-cis-retinoic acid, RA) at the dose of 40 mg/die for the treatment of acne vulgaris was the patient’s only ongoing pharmacological therapy since March 2021. Admission ECG showed sinus rhythm and diffuse repolarization abnormalities. Over the subsequent hours, the ECG monitoring revealed several non-sustained ventricular tachycardia (NSVT) episodes. A cardiac magnetic resonance (CMR) performed the day after admission confirmed the echocardiographic findings and revealed a LV circumferential mid-wall late gadolinium enhancement (LGE). A coronary computed tomography angiography (CCTA) ruled out coronary disease. Blood cultures, autoimmune panel, and genetic tests allowed for the reasonable exclusion of infective, autoimmune, or heritable causes of dilated cardiomyopathy (DCM). Absence of pre-existing coagulopathy was confirmed by a complete coagulation screening. The dosage of plasmatic homocysteine resulted in 11.2 µmol/L, slightly over the range of normality. Homocysteine, a sulfur-containing amino acid, is recycled into methionine by a transmethylation reaction requiring folate and vitamin B12. Several studies have highlighted that elevated plasma homocysteine concentrations are associated with an increased risk of premature occlusive vascular disease [1]. Liver dysfunction is not infrequently observed in patients treated with RA and can affect the function of cystathionine-b-synthase, an enzyme involved in homocysteine metabolism, causing hyperhomocysteinemia [1,2]. Other common adverse effects of isotretinoin are cheilitis, teratogenicity, myalgia, and arthritis; conversely, cardiac toxicity is not acknowledged as common [3,4,5,6]. During hospital stay, isotretinoin was stopped, and recommended heart failure therapy was set up. The management of renal infarction was a result of multidisciplinary consultations which led to a non-interventional approach and a close echographic follow-up of the hypo-perfused parenchymal dysmorphic renal lesion. The patient was discharged from our HUB hospital 12 days after admission and was referred to a cardiac rehabilitation program which allowed medical therapy titration. At the three-month follow-up visit, effort dyspnea was persistent, and echocardiography revealed a scanty improvement of the LV systolic function. The patient was thus re-admitted to our unit and underwent a subcutaneous implantable cardioverter-defibrillator (ICD) implantation and a comprehensive evaluation to be placed on the cardiac transplant list.

**Figure 2:**
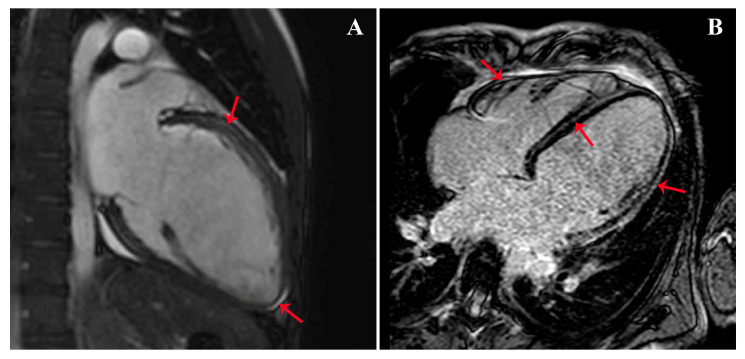
CMR with evidence of left ventricular circumferential mid-wall LGE (red arrows) in the long axis view (**A**) and 4-chambers view (**B**). Despite being deemed safe, isotretinoin treatment was reported to be possibly associated with the occurrence of DCM [7]; in the literature, two cases of myocarditis linked to tretinoin assumption were also described [8]. Furthermore, a potential connection between isotretinoin and thrombotic events, myocardial infarction (MI), and stroke were hypothesized [9,10]. In our case, the non-ischemic pattern of the LGE (this figure) is compatible with both DCM and myocarditis hypotheses. However, the normal erythrocyte sedimentation rate (ESR) and the absence of fever, symptoms of infection, and edema at CMR made acute myocarditis less likely. Based on the Naranjo algorithm [11], the patient’s clinical picture can be defined as a probable adverse reaction to isotretinoin use (adverse drug reaction probability score of 6). Recent studies demonstrated that RA plays a central role in the cardiac remodeling process following myocardial infarction by altering protein synthesis and cellular differentiation and proliferation [12,13]. RA supplementation has been linked to atrial and ventricular mass increase and significant LV dilatation [14]. These alterations are consistent with the myocardial remodeling consequent to myocardial injury or chronic hemodynamic overload and could support the hypothesis of a cause–effect relationship between RA treatment and the development of a DCM phenotype [14]. Thrombotic events occurring during the treatment with RA are rare, and despite being previously reported, the underlying pathophysiology remains mostly unexplained. In our patient, inherited causes of thrombophilia were excluded. Remarkably, cases of dysfibrinogenemia and vasculitis were reported in the course of treatment with RA [9,15]. Therefore, an RA-induced thrombotic diathesis cannot be excluded and represents, in the authors’ opinion, a deserving future investigation field. In conclusion, isotretinoin is among the most effective drugs for the treatment of severe acne vulgaris; however, in rare cases, it has been hypothesized to be possibly associated with potentially life-threatening cardiovascular adverse events in young and middle-aged patients. To the best of the authors’ knowledge, this is the first case of severe DCM and renal thromboembolism simultaneously occurring during isotretinoin treatment. Even if uncommon, the clinical relevance of these adverse events makes the investigation of potential cause–effect relations of great scientific and clinical interest.

## Data Availability

Not applicable.
